# Fundamental Studies of Rapidly Fabricated On-Chip Passive Micromixer for Modular Microfluidics

**DOI:** 10.3390/mi12020153

**Published:** 2021-02-04

**Authors:** Wenpeng Guo, Li Tang, Biqiang Zhou, Yingsing Fung

**Affiliations:** 1 First Affiliated Hospital of Shenzhen University, Shenzhen 518035, China; drzbq@hotmail.com; 2 The University of Hong Kong, Hong Kong, China; tangl@hku.hk (L.T.); ysfung@hku.hk (Y.F.)

**Keywords:** microfluidics, laser ablation, passive micromixer

## Abstract

Micromixers play an important role in many modular microfluidics. Complex on-chip mixing units and smooth channel surfaces ablated by lasers on polymers are well-known problems for microfluidic chip fabricating techniques. However, little is known about the ablation of rugged surfaces on polymer chips for mixing uses. This paper provides the first report of an on-chip compact micromixer simply, easily and quickly fabricated using laser-ablated irregular microspheric surfaces on a polymethyl methacrylate (PMMA) microfluidic chip for continuous mixing uses in modular microfluidics. The straight line channel geometry is designed for sequential mixing of nanoliter fluids in about 1 s. The results verify that up to about 90% of fluids can be mixed in a channel only 500 µm long, 200 µm wide and 150 µm deep using the developed micromixer fabricating method under optimized conditions. The computational flow dynamics simulation and experimental result agree well with each other.

## 1. Introduction

The mixing unit is a crucial element in most microfluidic chips and systems, such as the Micro Total Analysis System (µTAS) and Lab on a Chip devices. Usually, at this micrometer size scale and with liquids flowing relatively slowly, the flow is laminar and confluence liquids tend to flow side by side. Due to the importance of mixing in modular microfluidic chips, many excellent researchers have developed different mixing techniques, based on geometries that reduce diffusion length scales or induce secondary flow, incorporate miniature mixing balls or rods, utilize cross flows or alternating flow from the inlets, pulse one of the reagents, or apply electric, magnetic, or ultrasonic vibratory fields [1,2,3,4,5,6,7,8,9]. Generally, these mixing techniques can be broadly classified into two types: passive and active. In active mixing techniques, fluids are always perturbed by using an external energy source such as magnetic force, mechanical pulsation, electrohydrodynamic force, electrothermal force, acoustic vibration, and transverse electroosmotic force. In all of the external resources, transverse electroosmotic flow is a commonly used method to achieve efficient active micromixers. One of the main techniques used involves generating patterned transverse electroosmotic flow on heterogeneous surface charges with non-uniform, time-independent zeta potentials through on-chip integrated electrodes to disturb fluids [10,11,12]. However, the surface fabrication process is relative complex with regard to the method. One microfluidic on-chip mixing device was developed in which the electroosmotic flow at the two inlets pulse out of phase to enable the on-chip fluids mixing [12]. A T-form micromixer was used to perform electrokinetically driven mixing by use of switching electroosmotic flow operated either in a pinched mode or in a conventional switching mode [13]. The same scheme was also used to improve the on-chip mixing efficiency through the use of multiple streams flowing in a channel [14]. Despite the fabrication process of these types of micromixers being very simple, there are two major disadvantages. Firstly, the disturbed in-channel transverse flow is mostly 2D and it is difficult to deal with uniform mixing on the cross-section, especially in high depth ratio 3D channels. On the other hand, the flow remains a steady helical streamline and the stretching and folding of fluid is maintained in the same direction, resulting in the mixing being not very effective. A form of chaotic mixing was developed in microchannels via low frequency switching transverse electroosmotic flow generated on integrated microelectrodes to improve the mixing efficiency [15]. However all of these mixers need to integrate microelectrodes on chips to manipulate the electroosmotic flow, and electroosmotic flow can only be used for mixing. In passive mixing units, the flow field is perturbed by adding geometric obstacles or changing the channels geometry. Numerous researchers have reported various passive mixing methods involving the utilizing of chaotic advection, such as the zigzag, square-wave, rapid 3D passive rotation, staggered herringbone, and multivortex techniques. However, the present fabrication process of the active or passive mixing techniques is complex and external resources are always needed.

In this study, a simple and easily fabricated mixing method without the need of any complex or external resources was explored and a novel on-chip passive micromixer was fabricated and presented. Different sizes for the microspheric structures, relating to laser parameters in the mixing channel, were designed and verified at different mixing lengths and applied voltage potentials. To the authors’ knowledge, no such work is present in the literature. This newly fabricated on-chip passive micromixer can provide a real, simple method for on-chip sequential mixing and reduces the total analyzing time. This system has a good chance to be immediately applied in biochemical sample analysis as the total system is very mature and the operation is very simple.

## 2. Materials and Methods

### 2.1. Materials

Cetyltrimethylammonium bromide (CTAB), acetaldehyde, sodium hydroxide, and sodium tetraborate decahydrate were obtained from Sigma Chemicals (St. Louis, MO, USA). All chemicals and solvents were analytical grade and used without further purification unless otherwise stated. All the solutions were prepared in distilled deionized water unless otherwise stated. Stock borate buffer solution (40 mM), including 1 mmol/L CTAB, was prepared by dissolving the appropriate amounts into deionized distilled water and stored in a refrigerator at 4 °C. The working buffer was freshly prepared by mixing appropriate volumes of stock solutions with pH adjusted to 10.3 using 0.1 mol/L NaOH and an Accumet 910 pH meter (Fisher Scientific, Waltham, MA, USA). Prior to use, the buffer was filtered using a 0.45 µm Cole-Parmer non-sterile syringe filter with polytetra fluoroethylene (PTFE) membrane and degassed. Polymethyl methacrylate (PMMA) plates were acquired from RS Hong Kong.

### 2.2. Ablation of Microspheric Particles on PMMA Surface as Mixing Structures

The polymethyl methacrylate plate (30 mm × 30 mm × 1.5 mm) was placed into a CO_2_ laser machine (Venus series, FL, USA), which was controlled by a computer installed with CorelDRAW software. The plate surface was ablated under different laser powers and speeds to get different sizes of microspheric particles on the PMMA surface. The ablated PMMA plate was washed in an ultrasonic water tank and then blow-dried.

### 2.3. Numerical Simulations of Mixing Structures

**Figure 1:**
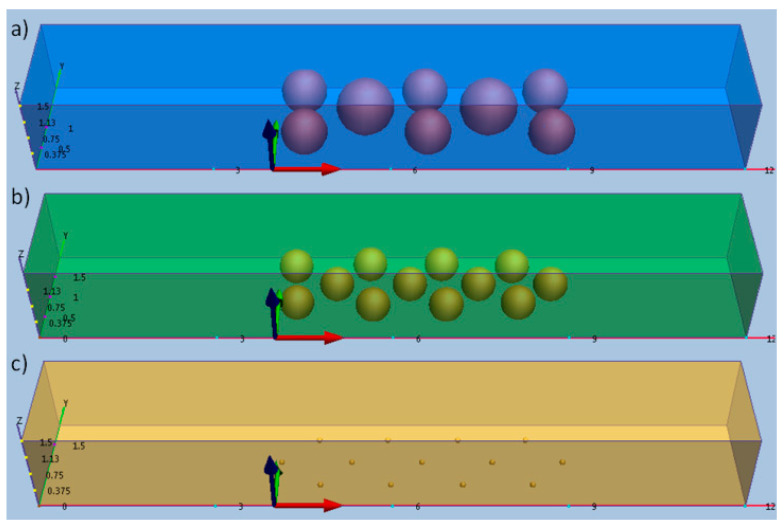
Three-dimensional schemes of the physical model with different sizes of microspheric structures under laser parameters: (**a**) 30% power and 60% speed (P3-S6), (**b**) 40% power and 50% speed (P4-S5), and (**c**) 40% power and 40% speed (P4-S4).

**Figure 2:**
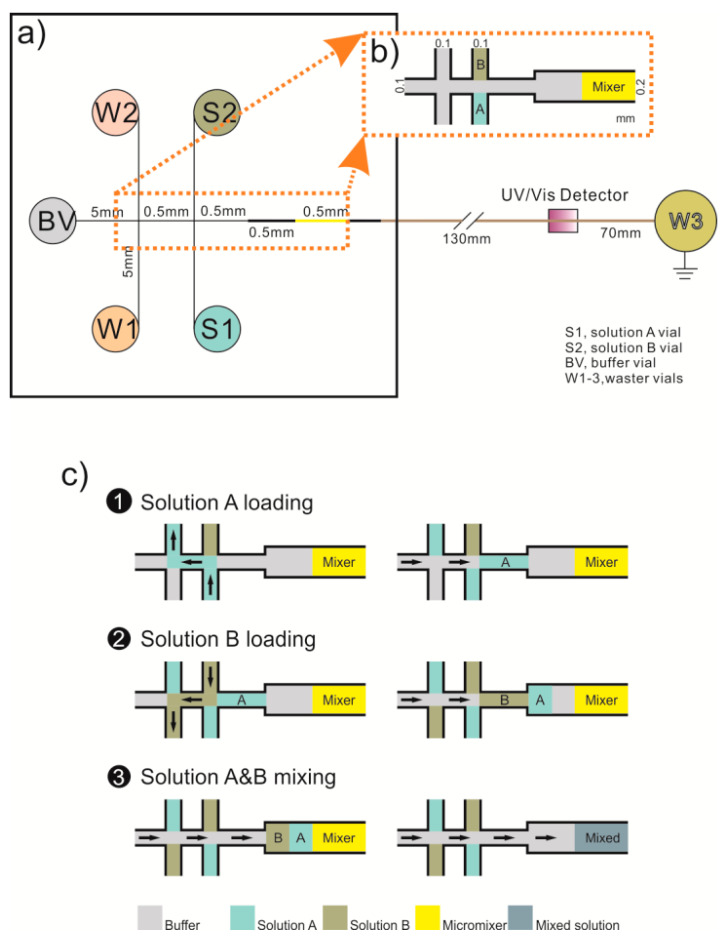
Schematic diagram showing (**a**) the developed polymethyl methacrylate (PMMA) microfluidic chip with (**b**) micromixer and (**c**) the sequential mixing steps.

Computational fluid dynamics (CFD) simulations of the flow field through the mixing channel were conducted to verify the results. The geometry used was the rectangle with irregular staggered microspheric formation. Three different sizes of microspheric structures were modeled and simulated (Figure 1). The rectangle channel was modeled with a length of 1200 µm, width of 200 µm, and a depth of 150 µm. The fluids flowed from left to right. The origin coordinate was set at a distance of 400 µm from the left inlet, which was also the start border of the mixing channel. The length for the mixing channel was 500 µm. The particle size for fabrication with 30% power and 60% speed (P3-S6) was modeled with 25% 100 µm diameter particles and for the rest with 80 µm diameters, as shown in Figure 2a. The particle size for fabricating with 40% power and 50% speed (P4-S5) was modeled with 100% 30 µm diameter particles, as shown in Figure 2b. The particle size for fabricating with 40% power and 40% speed (P4-S4) was modeled with 100% 1 µm diameter particles, as shown in Figure 2c. In calculating the flow velocity magnitude, the objects were modeled in three-dimensional and in 1 × 1 scales.

The incompressible viscous flow through the micromixer was modeled by solving the incompressible Navier–Stokes equations using a second-order projection method [16,17]. A 20 mM borate buffer at 25 °C was modeled with a constant viscosity of 0.914 cm^2^·S^−1^. We prescribed uniform electroosmotic mobility values of 0.473 × 10^−3^ cm^2^/Vs and a steady-state velocity of 0.142 cm/s at the inflow, which corresponds to a voltage potential of 300 V/cm. The computational domain extended upstream from the on-chip passive micromixer such that the steady-state velocity profile in the channel was developed prior to entering the micromixer section. The outflow was held at atmospheric pressure and standard non-slip conditions were imposed on all surfaces. A free tetrahedral mesh was used for the entire channel of the micromixer, with typical mesh elements being no larger than ~5 µm^3^ in volume for all the models studied.

### 2.4. Design and Fabrication of Micromixer with Irregular Microspheric Particles

A microfluidic chip with micromixer was made on a PMMA polymer base plate (30 mm × 30 mm × 1.5 mm) using the rapid prototyping technique, which involves the automatic construction of physical objects using solid freeform fabrication. It utilizes virtual designs from computer-aided design (CAD) or animation modeling software, such as CorelDRAW. The PMMA chip design and fabrication can be easily changed and realized with little cost and time using this technique. Two pairs of double-T microchannels, for introducing solutions, and one mixing unit were sculpted by micro-laser ablation under the control of the CAD software installed on one computer, as shown in Figure 2. 

The three irregular microspheric particles with different sizes on the mixing channel surface were ablated with 30% laser power and 60% speed, 40% laser power and 50% speed, and 40% laser power and 40% speed. Other channels were ablated with 30% laser power and 40% speed. The width of the mixing channel was about 200 µm, the length about 500 µm, the depth about 150 µm. The total volume was about 7.5 nL. For the double-T microchannel, the distance from the solution A vial (S1) and the solution B vial (S2) to the channel crossing was 5 mm. The double-T injector was 0.5 mm, which can be loaded with about 15 nL solutions.

A 20 cm capillary with 200 µm i.d. and an effective length of 13 cm was used to couple the PMMA microfluidic chip. The capillary was sandwiched between two fabricated PMMA plates and located on the center of the double-T microchannel. A thermally controlled hot plate press machine was used to bond the microchip under constant pressure and temperature set at 0.6 MPa and 92 °C, respectively, for 15 min. After cooling to room temperature, the bonded microfluidic chip was cleaned in distilled water in an ultrasonic bath and dried for the next use.

### 2.5. Dynamic Coating and Electroosmotic Flow Measurements on PMMA Microfluidic Chip

Laser-ablated PMMA chip channels were flushed with 0.1 mol/L HNO_3_ for 2 min and 0.1 mol/L NaOH for 5 min at 20 p.s.i. and then pretreated with deionized water for 10 min before use. The background electrolyte in all experiments was 20 mmol/L of borate buffer at pH 10.3, to which surfactants of the chosen concentration were added, if required. The buffer containing CTAB surfactants was used to flush the chip channel for 5 min at 1.0 p.s.i. In between runs, the PMMA chips were rinsed with deionized water for 1 min at 1.0 p.s.i.

The apparent electroosmotic flow (EOF) resulting from the coating of the channel surfaces was determined by the uncharged neutral marker method. Mesityl oxide was introduced as a neutral marker using a 2 s and 0.5 p.s.i hydrodynamic injection. Detection was at 254 nm. A constant voltage of −6 kV was applied. The EOF velocity (*ν_EOF_*) was determined from the migration time (*t*) using the effective length *L_d_* (the length from the inlet to the detector) of the total length *L_t_* (20 cm) of the capillary (Equation (1)):

(1) $$\upsilon_{EOF}=L_{d}/t$$

The magnitude of the electroosmotic mobility (*µ_EOF_*) was calculated using the migration time of mesityl oxide (*t_m_*), considering the applied electric field strength *E* (Equations (2) and (3)), where *V* is the applied voltage:

(2) $$\mu_{EOF}=\upsilon_{EOF}/E=\upsilon_{EOF}L_{t}/V$$

(3) $$\mu_{EOF}=L_{d}L_{t}/t_{m}V$$

### 2.6. Operation Procedure for the Microfluidic Chip with On-chip Sequential Mixing

Before initial use, the channels of the PMMA microfluidic chip were conditioned by sequentially adding 0.1 mol/L NaOH, distilled water, and deionized water for 5–10 min each through BV into the vials designated as S1/S2, and W1/W2/W3 (see below) using a pump placed at the end of the capillary. Prior to analysis, the channels were rinsed with 0.1 mol/L NaOH, distilled water, and deionized water for 2 min, and equilibrated with running buffer for 5 min. After being emptied, the buffer vials (BVs) and the waster cells (W1/W2/W3) were filled with 20 µL running buffer solution, respectively, followed by an addition of 10 µL of solution A into vial S1, then 10 µL of solution B into S2. After solution loading, a 1 min stabilization of the fluids in channel networks was undertaken, and then an electric field of the same magnitude (about −300 V/cm) was simultaneously applied in all the loading and separation channels.

The mixing threshold index (Mt index) in this study was 0.9, meaning that about 90% of fluid was completely mixed, and this was used to indicate the mixing efficiency. For each position, the mixing efficiency was the average value obtained by calculating sequence images with the same time interval, and the error bar on each figureshows the deviation range from the average value; when the value reached Mt, each fluid was assumed to be totally mixed.

The electrical configuration for conditioning and all experimentation involved application of −300 V to the S1/S2 vials and −6000 V to the BVs, with the W1/W2/W3 vials grounded. Following the above steps, 10 µL of solution A was loaded at S1 to replace the running buffer. Similarly, 10 µL of solution B was loaded at S2. Injection was performed for 10 s for solution A, then 10 s for the buffer and 10 s for solution B. After that, the running buffer was injected continuously. The experimental images were observed and recorded with a charge coupled device (CCD) camera attached to a microscope utilizing a 10× objective lens, with an image size of 512 × 512 pixels and a lateral resolution of 1 µm.

## 3. Results

### 3.1. Microfabrication and Surface Patterning of Mixer

**Figure 3:**
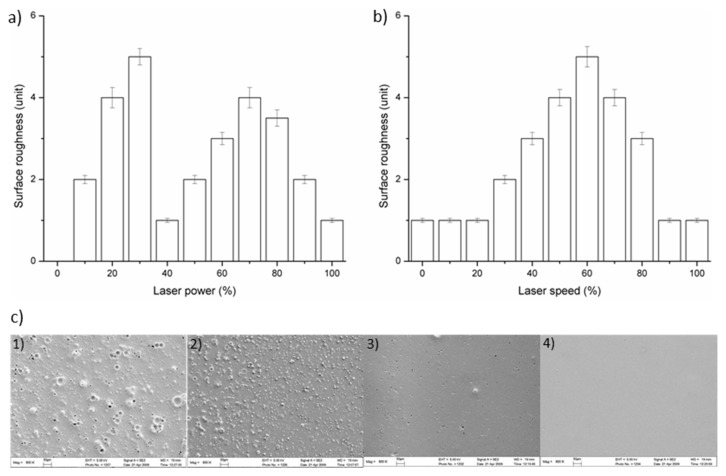
Laser ablation parameters for a PMMA chip surface were studied at different (**a**) laser powers and (**b**) laser speeds. The SEM images (**c**) show the surface patterns on PMMA under different laser power and speed ablating parameters. (**c1**) Laser power 30% and speed 60%, P3-S6; (**c2**) laser power 40% and speed 50%, P4-S5; (**c3**) laser power 40% and speed 40%, P4-S4; (**c4**) laser power 30% and speed 40%, P3-S4.

Generally, a smooth ablating surface is necessary for a chip, especially in the sample channel, because it can reduce the absorption of analytes to the surface. In this study, the feasibility and fabrication of a rough surface for mixing were demonstrated. The change of surface roughness depends on a combination of laser power and speed and here the surface rough unit (SRU) was defined by particles numbers as about 100 granules per 100 square micrometers on a PMMA chip surface ablated by a CO_2_ laser. The relationship between laser parameters and the SRU was investigated; the results for various percentages of laser power at 60% speed are shown in Figure 3a and for various percentages of laser speed at 30% power in Figure 3b. The results show that a rough surface can be easily acquired under different laser parameters. However, the size of the irregular particles produced by some ablating parameters was larger than 150 µm, which means that they overflowed the dimensions of the mixing channel. So, finally, three groups of parameters were investigated to find out which irregular particle sizes had the better effect on mixing efficiency. One group was fabricated with 30% laser power and 60% laser speed (P3-S6), as shown in Figure 3(c1). Another group was fabricated with 40% laser power and 50% laser speed (P4-S5), as shown in Figure 3(c2). The third was fabricated with 40% laser power and 40% laser speed (P4-S4), as shown in Figure 3(c3). The SEM image in Figure 3(c1) shows that the particle size for P3-S6 was ununiformed, on average, with 75% around 80 µm and others around 100 µm. Figure 3(c2) shows that the particles for P4-S5 mainly consisted of particles with 30 µm diameters. Figure 3(c3) shows that the particle sizes for P4-S4 were scattered, with some particles around 1 µm. Figure 3(c4) shows the smooth surface fabricated with 30% laser power and 40% laser speed (P3-S4). These results were used in the 3D modeling and CFD simulation.

### 3.2. CFD Simulation of Three Patterns of the Mixing Channel

To verify how the particle size affects the result of the mixing efficiency, the CFD simulation method was used in this study. Three groups, P3-S6, P4-S5, and P4-S4, were modeled with different packing particles size, as shown in Figure 1. In calculating the flow velocity magnitude the objects were modeled in three dimensions and in 1 × 1 scales. The incompressible viscous flow through the micromixer was modeled, with the 20 mM borate buffer at 25 °C modeled with a constant viscosity of 0.914 cm^2^·S^−1^. The average electroosmotic mobility values were prescribed as 0.473 × 10^−3^ cm^2^/Vs and the steady-state velocity as 0.142 cm/s at the inflow, which corresponds to a voltage potential of 300 V/cm. The computational domain extended upstream from the mixer so that the steady-state velocity profile in the channel was developed prior to entering the mixer section. The outflow was held at atmospheric pressure and standard non-slip conditions were imposed on all surfaces.

**Figure 4:**
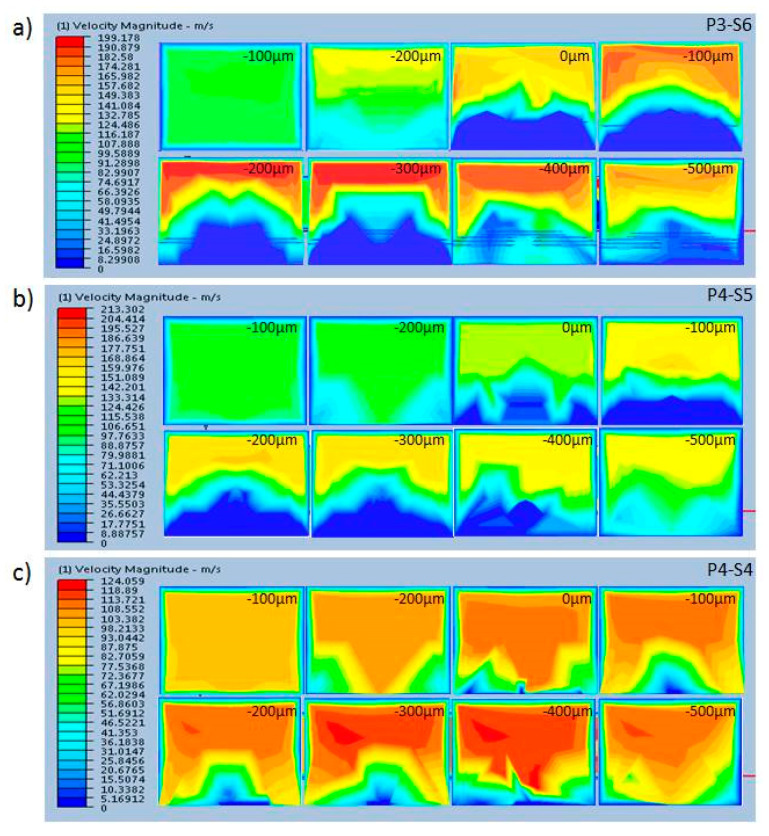
Schematic showing the distribution of velocity magnitude along the x-axis. (**a**) P3-S6, (**b**) P4-S5, and (**c**) P4-S4.

**Figure 5:**
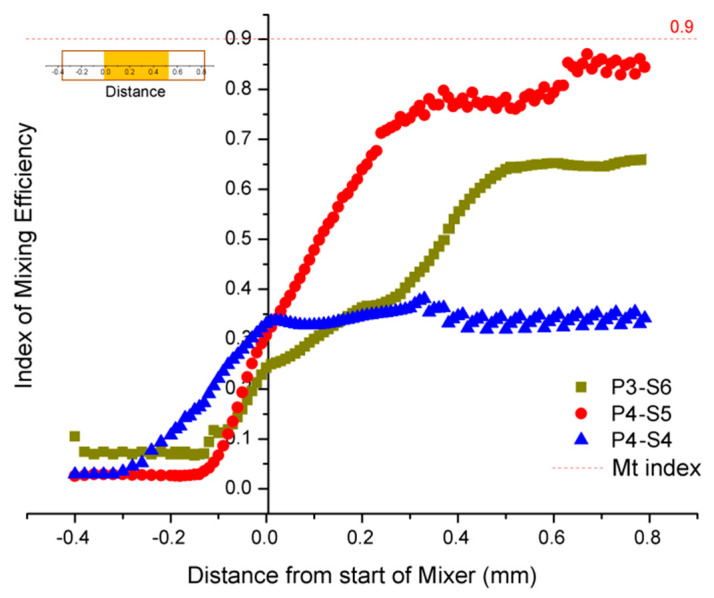
Computational fluid dynamics (CFD) simulations showing the distribution of velocity magnitude along the x-axis, which is generated by staggered microspheres in three different mixing channels.

The simulation results are shown in Figure 4 and Figure 5. These show that the mixing efficiency for P4-S5 nearly reached the Mt index, which means that about 90% of fluid was completely mixed. Another phenomenon observed was that there was about 30% of the mixing efficiency at the origin coordinate, which indicates that the fluids began to mix before flowing into the mixing channel.

### 3.3. Effect of Microchip Parameters on Mixing Efficiency

**Figure 6:**
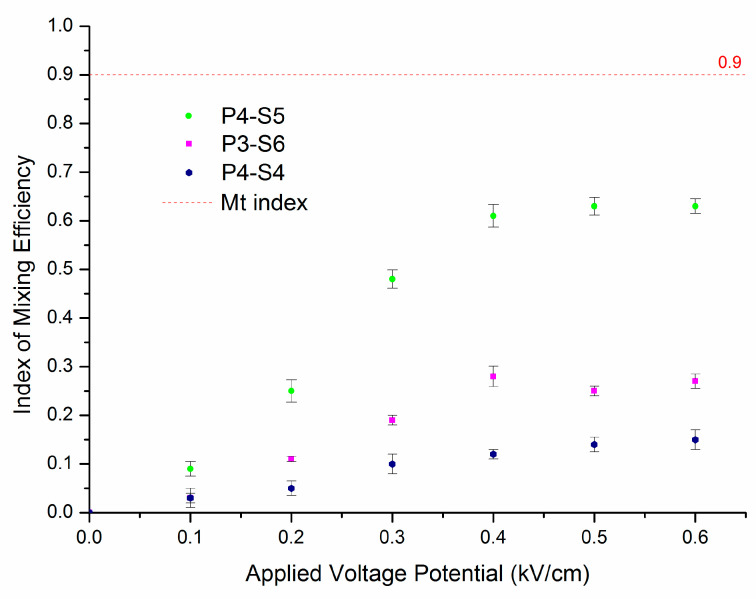
The index of the mixing efficiency with various commonly used potentials.

We next investigated the effects of microchip factors, such as applied potential, length of the mixing channel, and electroosmotic mobility, on the mixing efficiency under different fabricating parameters. The applied potential plays the key role in any mixing process using electroosmotic mobility. When the potential increases, the mobility of the EOF is improved and the flow circulation becomes faster in the mixing channel, which enhances the mixing efficiency. The mixing index of the captured cross-section images was measured in order to quantify the mixing efficiency, which was defined by *i* = 1−Is, where Is is the discrete intensity of segregation [15]. In a perfectly mixed system, Is = 0 or *i* = 1. Figure 5 shows the index of mixing efficiency at different applied potentials on a 500 µm length of mixing channel. For each position, the index of mixing efficiency *i* is the average value obtained by calculating sequence images with the same time interval; the error bar on the figures shows the deviation range from the average value. The mixing threshold index was 0.9 and it was assumed that fluids were completely mixed when the mixing index was reached Mt (the red dashed line shown in Figure 6). The Mt index in this study corresponded to 90% of the fluids being mixed. Figure 6 shows that the mixing efficiency for fluids was improved with increasing voltage. However, no completed mixing efficiency was found in commonly used potentials between 100–600 V/cm. We found that only 63% of the fluid was mixed for ablating parameters P4-S5 when the potential was increased to 400 V/cm. For the other parameters, P3-S3 only resulted in 28% of the fluid mixed and P4-S4 only 14% when the potential was higher than 400 V/cm. This indicates that the mixing efficiency became better when the potential increased from 100 V/cm to 400 V/cm without a lot of heat appearing. The potential 300 V/cm was the optimal frequency in this case. Figure 6 also demonstrates that the ablating parameter P4-S5 was better than P3-S6 and P4-S4.

**Figure 7:**
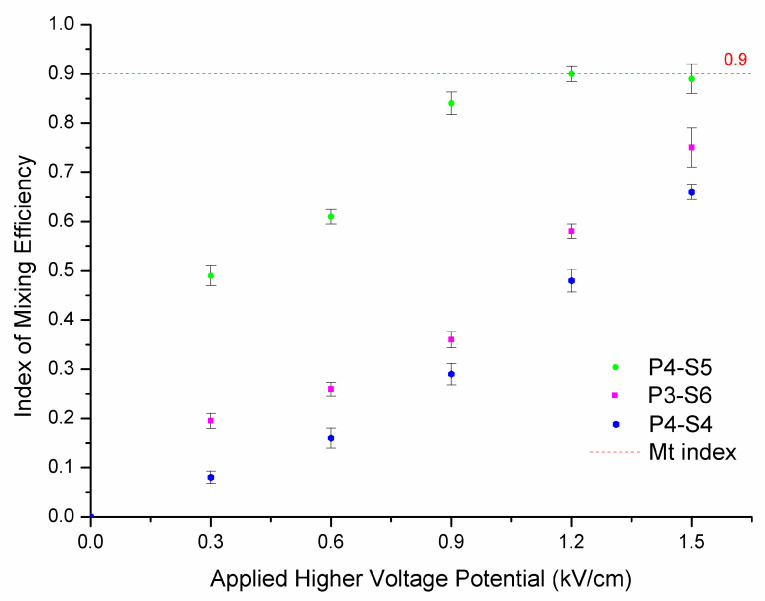
The index of the mixing efficiency with higher potentials.

Higher applied potentials were also investigated with regard to their relationships with mixing efficiency. Figure 7 shows the effect of higher potential on mixing efficiency when it is increased from commonly used levels below 600 V/cm to 1500 V/cm. The results show that fluid was mixed well when the potential was increased to 1000 V/cm for ablating parameter P4-S5. Although Figure 6 shows that the mixing efficiency for ablating parameter P4-S4 was not good for potential below 600 V/cm, the index of mixing efficiency reached around 70% when the potential was increased to 1500 V/cm, which is nearly fivefold higher than the 14% achieved at 600 V/cm. However, one issue cannot be ignored in capillary electrophoresis that use higher potential and that is heat. With the potential increased, abundant heat is inevitably generated. This may be the best explanation why mixing efficiency was still increased with ablating parameter P4-S5 even without improving the rough surface and electroosmotic mobility. During our experiments, some leakages of buffer from the microchip and air bubbles were observed when the potential was increased to 1200 V/cm. This indicates that the fluid was completely mixed when the higher potential was applied, rather than the reason for the improvement of mixing efficiency being related to the increased potential or electroosmotic mobility. It was the heat that made the molecules move faster and thus molecular heat diffusion helped to produce the results.

**Figure 8:**
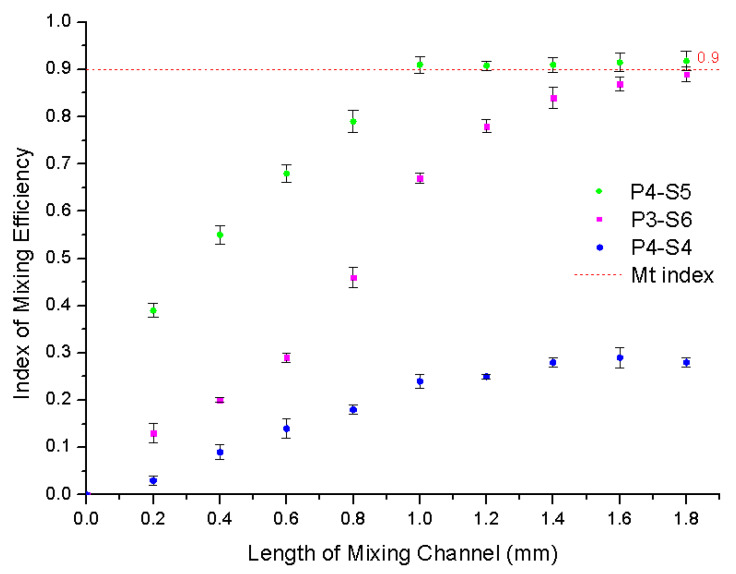
The index of the mixing efficiency with different lengths of the mixing channel.

The length of the mixing channel is another important factor during the mixing process, as the length is related to the mixing time of the fluids. Theoretically speaking, a longer mixing channel improves the mixing efficiency because it can increase the mixing time [13]. On the contrary, a shorter mixing channel decreases the mixing efficiency. However, a longer mixing channel increases the opportunity for uneven distribution of microspheric structures as well producing another problem, namely that with a longer mixing time inevitably comes an increase in the total analysis time. A comparison of mixing efficiencies using different lengths of the mixing channel under a constant applied power potential of 300 V/cm is shown in Figure 8. It shows that fluids were completed mixed when the length reached 1 mm for ablating parameters P4-S5, but only 65% of the fluid was mixed for ablating parameter P3-S6 and 23% for P4-S4. Mixing efficiency for P3-S6 increased rapidly when the length was increased from 1 mm to 1.2 mm and it reached the Mt index at 1.8 mm. The length of the mixing channel for P3-S6 was nearly onefold greater than for P4-S5 when the fluid was completed mixed. The results also show that increasing the length of the mixing channel for P4-S4 could only increase the mixing efficiency a little. This indicates that the on-chip mixing efficiency changed more effectively when the length of the mixing channel increased for P4-S5 and P3-S6 but not for P4-S4.

**Figure 9:**
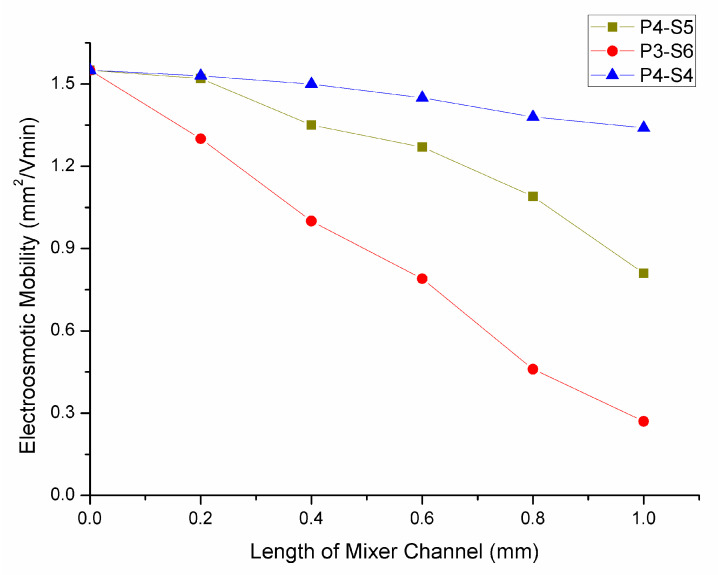
The electroosmotic mobility with various lengths of the mixing channel.

The variation of electroosmotic mobility in relation to the length of the mixing channel was also investigated since the staged channel can affect the electroosmotic mobility, which relates to the packed capillary used in electrophoresis. The laser-ablated rough surface in the mixing channel was similar, under varying conditions, to the different packing particle sizes in the capillary. Under the same buffer condition, if the particle size was larger, the electroosmotic mobility was reduced. So the problem was to find a balance between the decrease of electroosmotic mobility and the increase of channel length. Figure 9 shows the relationship between the electroosmotic mobility and the length of the mixing channel under a constant applied power potential of 300 V/cm. The results show that the electroosmotic mobility had a linear relationship with the length of mixing channel. The percentages by which the electroosmotic mobility decreased with an increase of 100 µm in mixing channel length were approximately 11% for P3-S6, 6% for P4-S5, and 2% for P4-S4. This result indicates that the particle size affected the linear relationship between the electroosmotic mobility and the length of the mixing channel. However, there was no linear relationship between the particle size and the decreasing percentages per 100 µm. A 500 µm length for the mixing channel was selected as the optimal mixing channel length after considering the increased mixing efficiency and the electroosmotic mobility.

**Figure 10:**
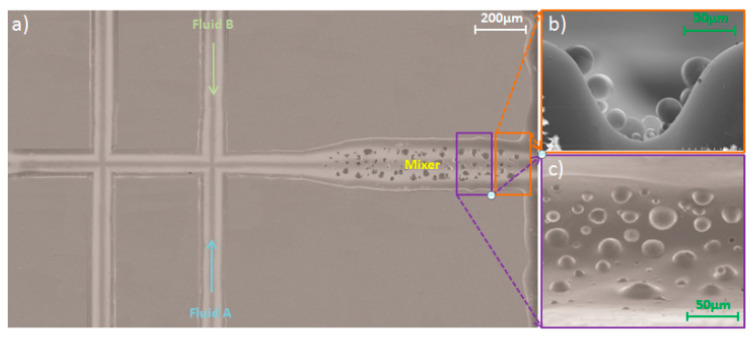
SEM images of the fabricated device showing (**a**) an overall view of the device and (**b**) showing the cross section of the ablated mixing channel; (**c**) showing the inner surface of the mixing channel. (length of mixing channel = 500 µm, applied potential = 300 V/cm).

**Figure 11:**
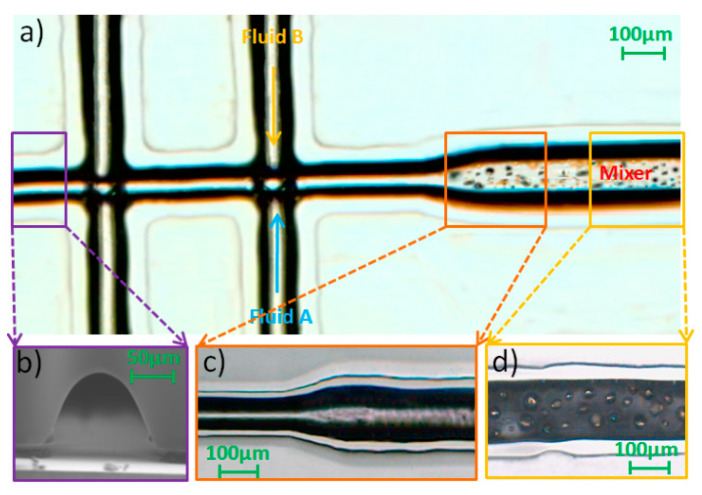
Microphotographs of the fabricated device showing (**a**) an overall view of the device; (**b**) the SEM image of the cross-section of the bonding channel; (**c**) the connection of the sample channel and mixing channel; and (**d**) the surface of the mixing channel. Length of mixing channel = 500 µm, applied potential = 300 V/cm.

**Figure 12:**
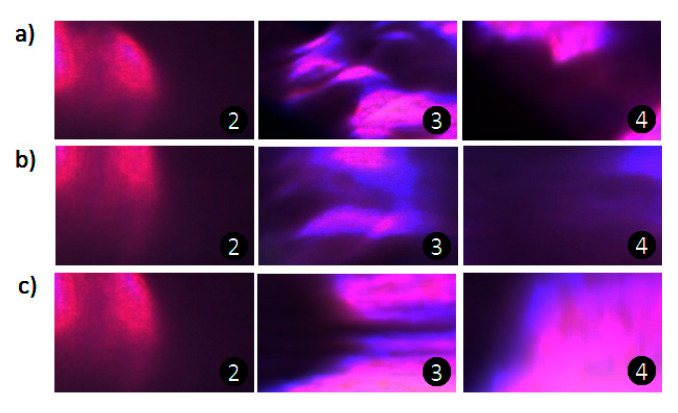
Images showing the mixing effects of the three types of mixing channel, with red dye used to indicate the effects before, in, and after the mixing channel for (**a**) P3-S6, (**b**) P4-S5, and (**c**) P4-S4. Length of mixing channel = 500 µm, applied potential = 300 V/cm.

The SEM image of the microfluidic chip used in this study is shown in Figure 10a. Figure 10b shows a cross-section of the mixing channel with particles and Figure 10c shows the surface of the mixing channel. Figure 11 shows a microphotograph of the fabricated microfluidic device. A red fluorescent dye was used to investigate mixing for three channel patterns related to different fabrication parameters. The results show that the device fabricated using parameter P4-S5 (Figure 12b) produced better mixing efficiency than the other two fabricating parameters.

**Figure 13:**
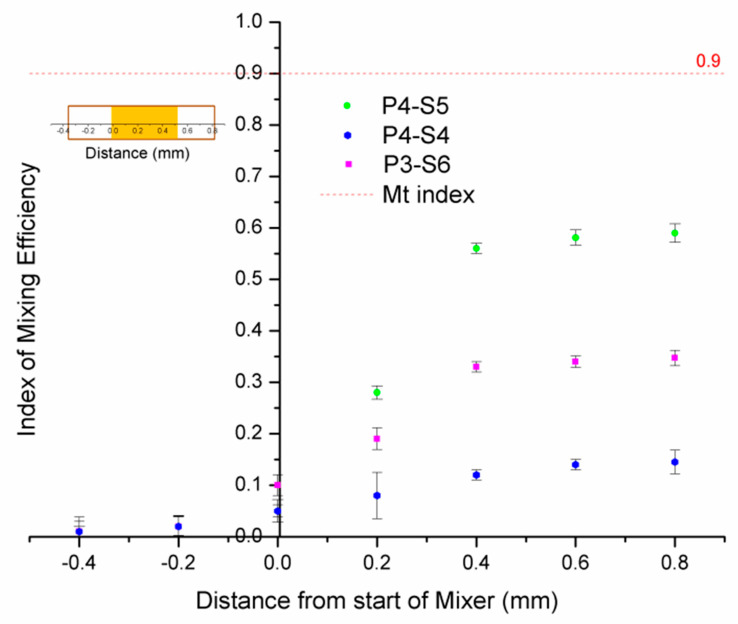
The index of the mixing efficiency before, in, and after the mixing channel. Length of mixing channel = 500 µm, applied potential = 300 V/cm.

To further verify the mixing performance of the mixing channel using different fabricating parameters, the fluids before mixing, during mixing, and after mixing were studied. This was the method used to examine whether the mixing channel worked or not when using the optimized potential and channel length. In this experiment, the fluids flowed from left to right and the flow direction was on the horizontal x-axis. The left border of the mixing channel was set as the origin of the coordinates and the right as the positive direction, as shown in Figure 13. The distance from the start of mixer was defined as the position of flow in relation to the origin of the coordinates. Figure 13 shows that mixing appeared at 200 µm when the fluids were driven forward by electroosmotic flow before moving into the mixing channel but the phenomenon was weak for all three of the fabricated chips. The mixing performance began to show a huge improvement when the fluids flowed into the mixing channel. However, the increase of mixing efficiency was not alike in the three different mixing channels. The fabricating method for P4-S5 showed the largest increase in efficiency—fivefold, from 10% to 60%—while for P3-S6 it increased by threefold and P4-S4 by 0.5-fold. This indicates that the fabricating method of the mixing channel contributes to the mixing efficiency more than self-mixing. 

The fabricating method for P4-S5 was the most suitable of the three methods for the fabrication of a mixing channel for on-chip continuous mixing, potentially improving the mixing efficiency by fivefold. The result is almost 50% lower than the simulation results shown in Figure 4. For the other two groups (P3-S6 and P4-S4), the same phenomena were found, except that the mixing performances of the three types of mixing channel agreed well with the results from the above CFD simulation analysis.

## 4. Discussion

In the present study, a simple on-chip passive micromixer, easily fabricated via the different sizes of the microspheric structure and ablated and controlled by a combination of laser-engraving parameters in the mixing unit, was investigated. No external resources or complex structure were used in this study.

**Figure 14:**
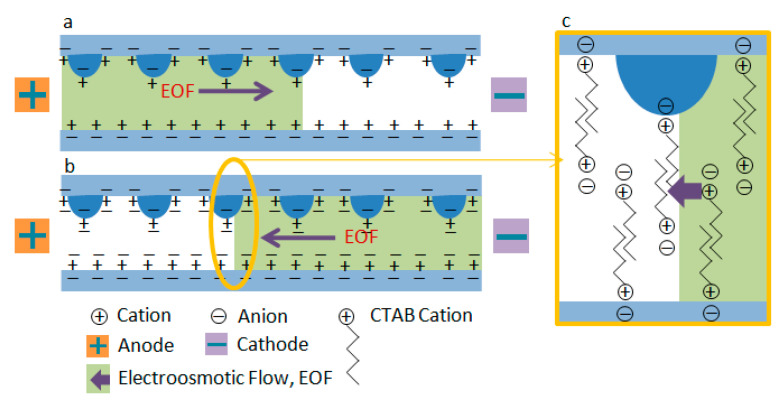
Schematic diagram showing the electroosmotic flow inside of the mixing channel on a PMMA chip. (**a**) Electroosmotic flow in the PMMA channel; (**b**) reversal of electroosmotic flow by addition of cationic surfactant CTAB to the buffer; (**c**) the bilayer formed inside the mixing channel.

It was found that the dynamic chemical modification and electroosmotic flow measurements on the PMMA microfluidic chip also played an important role in improving mixing efficiency, with the mixing structure ablated by a laser machine for the characters of the polymer. As is known, PMMA is a hydrophobic polymer material with a negatively charged surface for which a cathodic electroosmotic flow is usually observed, smaller than the electroosmotic flow in fused silica, as is shown in Figure 14a [18,19]. It was therefore expected that there would be hydrophobic interactions between the polymer surface and the surfactant but not electrostatic interactions, resulting in the introduction of positive charges and not only in a shielding of negative charges. Thus, a net positive surface charge could result without the need of aggregates or micellar structures. A similar mechanism (interaction via hydrophobic and hydrogen bonding) has been suggested for the interaction between neutral polymers and PMMA surfaces [20]. Conventionally, the mechanism by which single-chain cationic surfactants are adsorbed onto microchannel surfaces has been depicted as a bilayer, as in Figure 14b,c [21]. Previous studies from the colloid and interface science literatures have also suggested that bilayers were the predominant structure based on indirect evidence using adsorption isotherms [22]. In this experiment, the cationic surfactant CTAB was used as the additive for the borate running buffer. In the procedure, 0.1 mol/L HNO_3_ and 0.1 mol/L NaOH were first used to flush the channel at 20 p.s.i. to remove some sharp structures and then 20 mmol/L buffer with 0.5 mmol/L surfactants (CTAB) was flushed at 1 p.s.i. for 5 min to form a micellar surface and to provide dynamic EOF.

The results of the present study show that mixing efficiency was improved with the decrease of the injection length of solution and that the time was reduced to less than 1 s when the fluids were completed mixed (Table 1). The micromixer was fabricated using the P4-S5 method and the volume for the micromixer with a length of 0.5 mm length was about 30 nL. The applied potential was 300 V/cm. This result indicates that the solution volume in the mixing chamber affects mixing efficiency well for passive micromixer.

The results from our work demonstrate that the laser-ablated on-chip passive micromixer can be used for continuous mixing via EOF in PMMA polymer-based microfluidics without the need of any external resources. The I-form channel geometry, different from the commonly used T- or Y-forms, was designed for sequential mixing of nanoliter fluids in less than 1 s. The results verify that up to about 90% of fluids can be mixed with a channel length of only 500 µm, width of 200 µm, and depth of 150 µm using the developed micromixer fabricating method under optimized conditions. The experiment results agree well with the mixing result observed in the CFD simulation using a 500 µm length mixing channel. However, the potential shortcoming of the method is that the fabrication process may differ depending on the particular laser machine.

The results of the present study are very promising when compared to other related studies using complex on-chip structures for mixing procedures [15,23,24,25,26,27,28,29]. In some of these studies, external microelectrodes were used and integrated on the chip to generate frequency switching transverse EOF for chaotic mixing [15,24]. Other studies developed complex on-chip structures of T-, Zigzag, and Serpentine types [25,26]. Finally, some only presented the model design of the micromixer and showed the simulation results [27,28,29]. We intend to continue to study application of the described technique and fabrication method; the sequential mixing of nanoliter fluids is especially useful for processing biomedical samples with only nanoliter volumes using a microfluidic chip platform. Furthermore, other applications such as water purification based on modular microfluidics could make for interesting research [30].

## 5. Conclusions

In the present study, we investigated the parameters affecting the development of a simple, easily fabricated on-chip sequential mixing method and easily operable, low-cost device for modular microfluidics. This method has the potential to be immediately applied in biomedical sample analysis, as the total system is very mature and the operation very simple. Further research on the use of the developed method for biomedical applications will be conducted with a focus on adjustable mixing volume.

Based on the results, this proof-of-concept study supports the idea that this approach can be used to simply and rapidly produce on-chip compact passive micromixers using laser-ablated irregular microspheric surfaces on PMMA microfluidic chips for continuous mixing uses in modular microfluidics. As there is no need for a complex on-chip structure or external forces, this passive micromixer can be used as a stand-alone device or solo modular microfluidic part.

## Figures and Tables

**Table 1:** The mixing parameters for loading different volumes of solution.

Plug A (Length)	Plug B (Length)	Plug A + B (Volume ^a^)	Mixing Parameters			
			Efficiency ^b^	RSD ^c^	Time ^d^	RSD ^c^
1 mm	1 mm	~30 nL	0.05	1.5%	3.12 s	3.24%
0.9 mm	0.9 mm	~27 nL	0.12	1.8%	2.87 s	3.67%
0.8 mm	0.8 mm	~24 nL	0.24	1.9%	2.43 s	3.75%
0.7 mm	0.7 mm	~21 nL	0.38	2.3%	1.91 s	4.10%
0.6 mm	0.6 mm	~18 nL	0.51	2.5%	1.24 s	3.89%
0.5 mm	0.5 mm	~15 nL	0.62	2.6%	1.03 s	4.51%
0.4 mm	0.4 mm	~12 nL	0.70	2.4%	0.97 s	4.07%
0.3 mm	0.3 mm	~9 nL	0.79	2.2%	0.93 s	4.22%
0.2 mm	0.2 mm	~6 nL	0.87	1.3%	0.89 s	4.91%

Note: ^a^ the cross-section was assumed to be a semicircle, with radius = 0.1 mm and volume = (πr^2^/2) × length; ^b^ index of efficiency, 0.9 was assumed as completely mixed; ^c^ RSD = relative standard deviation (*n* = 3); ^d^ time began with the completion of the sequential injection of solutions A and B and ended after all solutions had passed through the micromixer.

## Data Availability

Data is contained within the article.
